# Stochastic Adder Circuits with Improved Entropy Output

**DOI:** 10.3390/e25121592

**Published:** 2023-11-28

**Authors:** Mateja Batelić, Mario Stipčević

**Affiliations:** 1 Department of Physics, Faculty of Science, University of Zagreb, Bijenička Cesta 32, 10000 Zagreb, Croatia; 2 Photonics and Quantum Optics Unit, Center of Excellence for Advanced Materials and Sensing Devices, Ruđer Bošković Institute, Bijenička Cesta 54, 10000 Zagreb, Croatia

**Keywords:** random pulse computing, stochastic computing, stochastic adder, circuit entropy, random flip-flop, quantum randomness

## Abstract

Random pulse computing (RPC), the third paradigm along with digital and quantum computing, draws inspiration from biology, particularly the functioning of neurons. Here, we study information processing in random pulse computing circuits intended for the summation of numbers. Based on the information-theoretic merits of entropy budget and relative Kolmogorov–Sinai entropy, we investigate the prior art and propose new circuits: three deterministic adders with significantly improved output entropy and one exact nondeterministic adder that requires much less additional entropy than the previous art. All circuits are realized and tested experimentally, using quantum entropy sources and reconfigurable logic devices. Not only the proposed circuits yield a precise mathematical result and have output entropy near maximum, which satisfies the need for building a programmable random pulse computer, but also they provide affordable hardware options for generating additional entropy.

## 1. Introduction

The concept of entropy was initially introduced by the German theoretical physicist Rudolf Julius Emanuel Clausius in 1865 [1]. Entropy has various definitions, with the most well known being the thermodynamic and statistical interpretations. However, this paper centers around the concept of entropy in information theory, specifically the Kolmogorov–Sinai entropy, named after the Russian mathematicians Andrey Nikolayevich Kolmogorov and Yakov Grigorevich Sinai.

In this context, the entropy of an information system aligns with the entropy of a physical system, represented by logical circuits, notably Boolean algebra, which forms the foundation of modern digital computers. Beyond digital computing, we now recognize two other computing paradigms: quantum computing and random pulse computing. It is precisely the latter paradigm that captures the focus of this paper.

Random pulse computing, introduced by John von Neumann in 1956 [2], stands out among computing paradigms because of its unique approach to data representation. While digital computers rely on bits as the smallest unit of data, and quantum computers use qubits, a random pulse computer (RPC) employs a different type of data: a random sequence of pulses called random pulse train (RPT). In this sequence, pulses occur unpredictably over time, mirroring the spontaneous nature of nerve impulses in living organisms. The primary parameter for this random pulse train is the pulse rate, precisely the pulse probability, expressed as a real number within this paradigm.

The RPC paradigm, also known as stochastic computing (SC) [3,4,5], enjoyed popularity until the 1970s when digital computing took the forefront. Renewed interest emerged in the second decade of this century [6,7]. Today, stochastic computing is instrumental in addressing challenges that digital computers face in terms of execution time, hardware requirements, or energy consumption. These challenges encompass advanced tasks such as deep learning, aiming to replicate the workings of the human brain, as well as image processing applications like edge detection and noise reduction [8].

The recent boost of interest in this computing paradigm is primarily motivated by the seamless processing of data in a continuous stream, enabling the attainment of results at maximum speed without the prerequisite of prior data accumulation, as is customary in digital computers. It seems particularly well suited for applications requiring massive parallelism and/or fault tolerance [9], such as artificial intelligence.

Naturally, the ultimate processing speed is contingent on the capabilities of the hardware. As computer hardware advances, it yields progressively faster and more precise results without necessitating improvements to the method itself. Furthermore, owing to the stochastic nature of the signals, the bulk of the information arrives initially, followed by gradual corrections as the statistical error diminishes with the square root of the time. Hence, this presents another compelling and robust rationale for the remarkable efficiency and utility of RPC.

The field of random pulse computation is still in development. Unlike the Boolean algebra that governs the functionality of a digital computer, an analogous theoretical framework for a random pulse computer is still evading researchers. Even though attempts have been made towards a general functional synthesis via expansion into Bernstein polynomials [9] or via a spectral transform approach [10], these cannot be automated since they require creative guesswork and/or complex optimizations. Recently, promising techniques of approximation of functions by linear or low-order polynomial parts are being developed [11,12].

However, the problem that is stalling the field of RPC computation is loss of computational accuracy in chained operations. Namely, RPC circuits function under the assumption of receiving perfectly random and statistically independent input streams [13,14]. However, even under that assumption, some circuits output a correlated output that is unsuitable for further calculations. Various techniques of correlation management have been proposed, such as delay-line decorrelation [15] and stochastic-to-digital and digital-to-stochastic conversion (isolation) [13], unfortunately with limited success while introducing elevated hardware complexity, higher energy consumption, and prolonged execution time costs. Attempts towards using correlations to an avail faced complications in achieving and maintaining the desired type and level of correlation [16,17]. In recent years, the development of new RPC circuits has been oriented more towards correlation-tolerant applications in image processing [18,19,20], sound processing [21], and neural networks [22], where correlations play a less detrimental role than in numerical computation.

In an effort to quantify and pinpoint the source of correlations in RPC, in our previous publications, we have pioneered concepts of *random flip-flop*, *digital random frequency synthesis* [23], *entropy budget*, and *relative entropy* [24]. In the latter, we presented several new circuits for division, subtraction, and comparison of numbers, offering different trade-offs between output randomness, circuit complexity, and precision. In this study, we employ these new tools in order to gain new insight into the inner workings of RPC circuits specific to the arithmetic operation of addition. We will investigate and compare several summing circuits: two already known in the art and five presented here for the first time.

## 2. Theoretical Background

**Figure 1:**
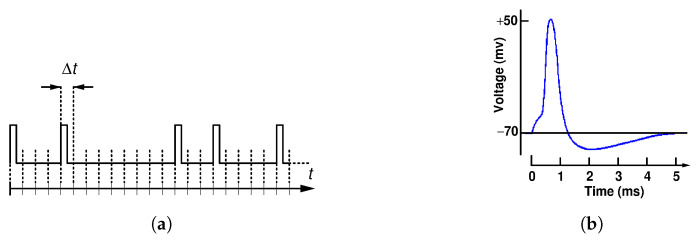
Random pulse train (RPT) with discrete pulse intervals of duration Δt, where each pulse is an independent binomial random event with the probability *p* (**a**); a complete cycle of a mammalian nerve pulse (**b**).

Random pulse computing relies on sequences of random pulses. A random pulse train (RPT) consists of a series of square electrical pulses with uniform and fixed width and height placed within discrete time intervals of a constant duration Δt, which is in fact the period of the system clock (CLK). All RPTs in the system are synchronized to CLK. An illustrative example of an RPT is shown in Figure 1a. The discretization of the time axis enables pulses to be systematically organized over time, while creating a pattern akin to the electrical impulses observed in mammals, as depicted in Figure 1b. It also enables us to represent the whole RPT as a sequence of bits by assigning the value of 1 to an interval containing a pulse and the value of 0 to an interval containing no pulse. This numerical representation of RPTs is crucial for our analysis of entropy.

This physical realization of an RPT is named *unipolar* and in the random pulse computer represents the number *p*. Other realizations of an RPT, much less frequently in use, include *bipolar* and *inverted-bipolar* [10], which differ from unipolar in that they represent a value 2p−1 or 1−2p, respectively, while having the exact same waveform. These realizations cover numbers in the range [−1,1]. Other realizations have been investigated too [5,7]. Even though there is a one-to-one correspondence between all of these realizations, the information processing circuits for the same function are surprisingly different. In this work, we adopt the by-far most frequently used unipolar representation of an RPT, because it closely resembles the electrical impulses in mammalian nerve cell synapses and is easily realized in the convenient reconfigurable field-programmable gate array (FPGA) technology.

In this study, unlike in virtually all others, where randomness is created by a pseudo-random process, for example, in Refs. [7,9,11,15,22], we use randomness originating from the quantum process of emission of a photon from a light-emitting diode (LED) and its subsequent detection by a single-photon detector, which we call quantum entropy source. Namely, inside the LED diode, two semiconductor layers coexist. The conductivity of these semiconductors is augmented through doping, leading to the creation of p-type and n-type semiconductors, contingent on the concentration of valence electrons introduced as impurities. These layers combine to form a p–n junction, where “holes” from the p-type intermingle with “electrons” from the n-type, giving rise to a depletion region. When the diode is forward-biased, electrons from the conduction band pair with holes from the valence bands, which leads to the emission of photons. While an average intensity depends on biasing, the creation of each photon is a random Poissonian process. This inherent randomness is crucial for the execution of subsequent experiments. The quantum effect observed during photon detection is the photoelectric effect, where light of constant macroscopic intensity impinges upon a single-photon avalanche diode (SPAD). This approach to obtaining signals from a random source was chosen due to the fact that the Poissonian process maximizes entropy, which is the central theme of this work.

We were the first to introduce quantum randomness in RPC [23], in the form of the so-called *random flip-flop* (RFF). The rationale behind that is to avoid complex management of seeding many pseudo-random sources, needed in RPC, and taking measures they are not mutually correlated. Indeed, each quantum entropy source is random from the moment it is turned on, without any seeding, and it is completely uncorrelated with anything else in the universe. Recently, a group of scientists from EPFL pioneered the chip, made in a standard 55 nm Si-CMOS process that contains 2800 independent RFFs [25], thus demonstrating the viability of that approach.

An RPT sequence is characterized by a constant pulse probability p∈[0,1], which is representing the likelihood of a pulse occurring in the subsequent discrete time interval. This real number, *p*, is the numerical value carried by the RPT in the random pulse computer. An RPC circuit typically receives one (or more) input RPT and produces one output RPT. In passing, we note that this topology is reminiscent of a mammalian neuron, which also features multiple inputs and a single output.

To arrive at an information-theoretic criterion for the evaluation of RPC circuits that will be used throughout this study, let us first remind ourselves of the Shannon entropy of a long sequence of bits, for symbols (patterns) n≥1 bits long, so called n-grams:

(1) $$H_{n}=-\sum_{i=0}^{2^{n}-1}p_{i}log_{2}\left(p_{i}\right).$$

where $p_{i}$ is a probability of finding the *i*-th n-gram in the sequence. The Kolmogorov–Sinai entropy h(x), defined as

(2) $$h\left(x\right)=\lim_{n\to \infty }\frac{H_{n}\left(x\right)}{n},$$

is interesting because it quantifies entropy per bit generated by an entropy source. In our case, we use it to estimate the entropy generated by an RPC circuit. Now, let us imagine that an RPC circuit outputs a perfectly random RPT with a small pulse probability *p*, say 0.1. Then, the Kolmogorov–Sinai entropy will be small too and will not nearly reach unity. This shows that this entropy by itself is not a good measure of the quality of an output of an RPC circuit. Instead, we introduced in Ref. [24] the *relative Kolmogorov–Sinai entropy*, or *relative entropy* for short (not to be confused with Kullback–Leibler divergence), which quantifies the extent to which the entropy of an RPT compares with the maximum entropy it could have as a binomial process, given its pulse probability. We define it as

(3) $$H_{rel}=\frac{h(x)}{h_{1}\left(x\right)},$$

where $h_{1}\left(x\right)=H_{1}\left(x\right)$ for a binomial process [24]. As will be discussed, in the construction of circuits, we aim that the output of an RPC circuit features relative entropy as close to unity as achievable.

**Figure 2:**
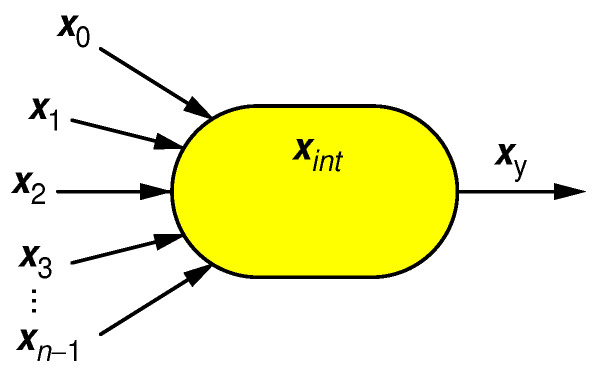
Possible entropy sources available for generating output random pulse train $x_{y}$ are input strings $x_{0},\ldots ,x_{n-1}$ and internal entropy source(s) $x_{int}$.

The second tool we are going to use is the *entropy budget criterion* (EBC). It applies to any RPC circuit, generally portrayed in Figure 2.

The EBC states that the entropy of the output RPT of a general RPC circuit, shown in Figure 2, may not exceed the total entropy of all input RPTs and all internal entropy source(s) [24]:

(4) $$H\left(p\left(x_{y}\right)\right)\leq h\left(x_{int}\right)+\sum_{i=0}^{n-1}h\left(x_{i}\right),$$

where $x_{i},i=0,\ldots ,n-1$ symbolize the input RPTs, $x_{int}$ represents all internal source(s) of entropy, the output RPT is presented by $x_{y}$, and $p(x_{y})$ stands for its pulse probability. Finally, $H(p\left(x_{y}\right))$ is defined as a single-variable function equivalent of the Shannon entropy for 1-bit n-gram:

(5) $$H\left(p\right)=-plog_{2}\left(p\right)-\left(1-p\right)log_{2}\left(1-p\right).$$

A design of an RPC circuit involves combining pulse probabilities of input RPTs via laws of probability, with the purpose of performing a desired mathematical operation. For example, ANDing two input RPTs having pulse probabilities $p_{0}$ and $p_{1}$ would yield an RPT with a pulse probability $p_{Y}=p_{0}p_{1}$, provided that the input RPTs are mutually statistically independent and that each of them is random, i.e., has no autocorrelation. Thus, a single AND gate may serve as an RPC circuit that performs the multiplication of two real numbers. More complex calculations are obtained by the networking of RPC circuits, which implies that outputs of some are used as inputs to the other circuits. This necessitates that not only inputs but also the output RPT of an RPC circuit are a result of a random binomial process and, as such, free of any autocorrelation. The Kolmogorov–Sinai entropy of Equation (2) is an appropriate measure of the required property. Because of its limes into infinite-length n-grams, any autocorrelation whatsoever would result in an entropy value lower than without the autocorrelation. Normalized to the interval [0,1], the relative entropy of Equation (3) is thus an appropriate merit of the statistical quality of the output of an RPC and will serve as a basis for the evaluation of circuits investigated here.

Regarding the possible entropy source(s) within the circuit, circuits are classified into two main categories: deterministic and nondeterministic. Deterministic circuits are those devoid of any supplementary entropy sources beyond the entropy inherent in their input sequences, namely, for which $h(x_{int})=0$. Conversely, nondeterministic circuits integrate extra entropy sources, such that $h(x_{int})>0$.

In this work, the internal source of entropy $x_{int}$ takes the form of a random flip-flop (RFF), which in turn employs its internal RPT of quantum origin to secure its proper operation, as will be explained.

## 3. Materials and Methods

The functional research of RPC circuits can be divided into four approaches: (a) functional simulation of a circuit (in software) based on its detailed or block schematics, (b) simulation of a circuit at the level of the computer-aided chip design tool, (c) performing experiment(s) on an actual physical circuit realized in an application-specific integrated circuit (ASIC), and (d) performing experiment(s) on an RPC circuit built within an FPGA. In this study, we use quantum randomness both for the preparation of random variables and for the internal entropy of RPC circuits, which is a rather novel approach. Therefore, we decided to test circuits experimentally. Circuits are initially designed using method (a), and then implemented and tested using method (d).

**Figure 3:**
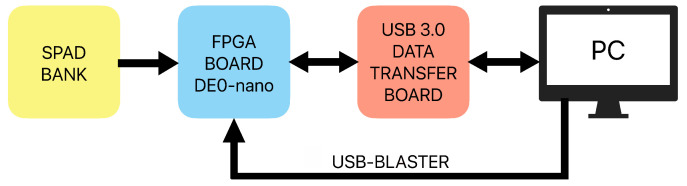
The experimental setup for the testing of random pulse computing circuits comprising quantum entropy source (SPAD Bank), an FPGA board on which RPC circuits are implemented, a fast FTDI-based data transfer board, and a PC computer for the programming of the FPGA, conducting automated measurements, and for offline data analysis.

The experimental setup used in this study, described in detail in Ref. [24], is shown schematically in Figure 3.

It comprises a PC computer, a bank of home-made LED-illuminated SPAD-based single-photon detectors [26] used as a quantum entropy source (SPAD bank), the Altera/Intel Cyclone IV field-programmable gate array (FPGA) on a DE0-Nano educational board from Terasic [27] in which the RPC circuitry is realized, and a home-made Future Technology Devices International Ltd, Glasgow, United Kingdom (FTDI)–based fast data transfer board capable of the simultaneous transfer of 16 RPTs, in real time, into the PC via a USB 3.0 port. The PC computer runs the Quartus 17.0 suite for programming the RPC circuits’ firmware into the FPGA through the USB-Blaster serial interface. It also runs a suite of home-made programs for experiment steering and data analysis.

With this setup, we were able to program arbitrary RPC circuits into the FPGA, put them under an automated test conducted by the PC computer, and collect all input and output data, as well as intermediate data if needed, to the PC computer memory (solid-state disc) for offline analysis. In particular, we were able to estimate the relative entropy from that data. In our approach, as explained before, all time intervals of a measured RPT are saved into the PC memory in the form of a long sequence of bits: value of 1 for an interval containing a pulse and value of 0 otherwise. Thus, a sequence of physical pulses in the FPGA is represented by a sequence of bits in the computer.

In addition to the setup described above, two benchtop instruments were employed in this experiment to check the correctness of the implementation of circuits into the FPGA chip, specifically the timewise alignment and integrity of internal waveforms and signals. These instruments, namely, the oscilloscope Rigol model MSO5204 and the frequency counter Hameg model HM8123, played a crucial role in identifying and removing any bugs in the actual physical implementations of RPC circuits.

### Generation of RPTs

It has been observed that the impulse sequences generated by human sensory neurons and in the human brain bear a striking resemblance to RPTs [28]. This intriguing similarity has sparkled research in biomimetic systems centered around the processing of RPTs. Since the only information carried by an RPT is its pulse probability, a valid question arises: how can this probability be generated with high precision?

In a realistic application of a random pulse computer, input RPTs would be generated by a sensory system, comprising, for example, a camera utilizing an array of photon-counting pixels [29], pressure detectors, microphones, etc. Nevertheless, the RPC that would process the data requires in itself the generation of multiple RPTs, sometimes with a precise pulse probability needed for computational constants. Below, we explain how such RPTs are generated for the purpose of this study.

Following a photon detection, SPAD detectors generate an electrical impulse of a square waveform, precisely the required shape for this experiment. Photon detection exhibits the exponential waiting time probability density distribution, cut off at times below the detector’s dead time whose duration is negligible in comparison with the basic CLK interval Δt of 1 μs. The intensity of light falling upon the detector is kept constant so that the process of generating a sequence of single-photon detections is stationary and provides consistent results over time.

For the generation of an RPT of any desired pulse probability, we used a digital random frequency synthesizer (DRFS) approach with multiple illuminated single-photon detectors serving as a source of randomness [23]. A DRFS circuit consists of an arbitrary number *N* of RFFs and can generate an RPT with a pulse probability *p* of magnitude in the interval [0,1], in $2^{N}+1$ equally spaced steps using a binomial process, which thus becomes the fundamental process in this experiment. To reach an absolute precision of 0.1%, we used N=10 throughout this study. With two DRFSs, we were able to generate two mutually independent RPTs with arbitrary probabilities, henceforth denoted as $p_{0}$ and $p_{1}$, which were used as input variables to the tested RPC circuits.

## 4. Results

In this work, the influence of entropy was investigated in circuits that perform the operation of the addition of two numbers. Because the plain addition of two probabilities, $p_{0}$ and $p_{1}$, could result in a value greater than 1, which is impossible to represent by an RPT, the idea is to use a circuit that would calculate the addition operation given by

(6) $$p_{y}=\frac{p_{0}+p_{1}}{2}.$$

This mathematical operation is technically a “half-addition”, and is indeed sometimes referred to by that name in the literature. Since in this work we only deal with that operation, we will henceforth simply call it “addition”. This operation is “closed” in the sense that, for any possible input values in the interval [0,1], its value will stay within the same interval.

However, by virtue of the EBC, an exact deterministic circuit for adding two probabilities according to Equation (6) may not exist [24]. The EBC entropy budget of any two-input deterministic RPC circuit is given by

(7) $$H\left(p_{0},p_{1}\right)=H\left(p_{y}\right)-\left(H\left(p_{0}\right)+H\left(p_{1}\right)\right).$$

**Figure 4:**
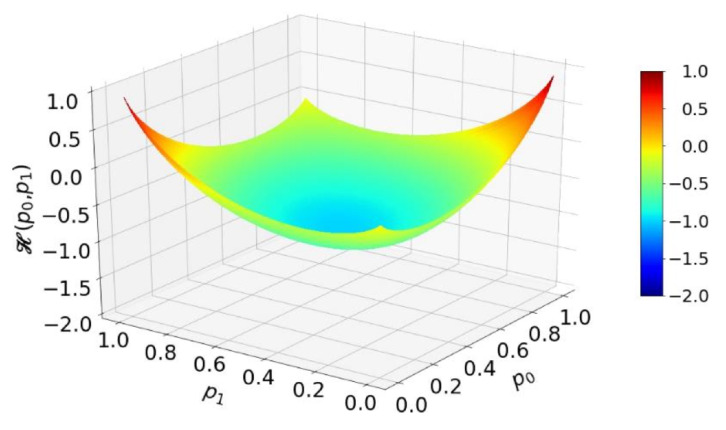
The entropy budget of an addition circuit, with the input probabilities $p_{0}$ and $p_{1}$, performing the operation given in Equation (6).

Figure 4 shows that, for a certain range of the input values $p_{0}$ and $p_{1}$, the total entropy available from inputs is greater than the amount of entropy required to generate an output RPT, indicated by the entropy budget being less than zero. There is no restriction on performing the required operation from the point of entropy in that region of input probabilities.

On the contrary, in regions of orange and red tongues, the total input entropy is insufficient to generate the output RPT, and that means that no circuit can perform the addition unless it either contains or can receive an additional entropy. Consequently, there is no exact deterministic circuit for the addition operation of Equation (6).

**Figure 5:**
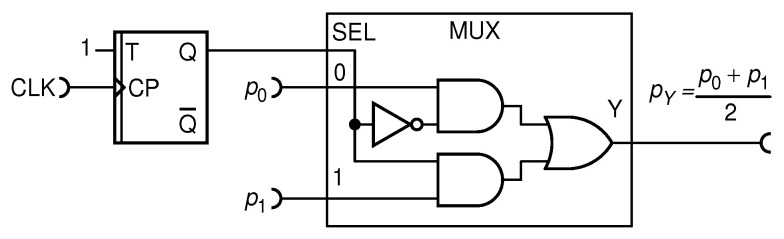
Scheme of an addition circuit using a multiplexer (MUX) and T random flip-flop (TRFF). The values $p_{0}$ and $p_{1}$ are input probabilities, while $p_{Y}$ is the output result.

Nonetheless, a nondeterministic circuit that performs approximate addition is possible. Probably the simplest one is shown in Figure 5.

Basically, this addition circuit samples pulses from each input RPT with a probability of 1/2 and conveys pulses to the output Y, resulting in the mathematical operation given in Equation (6). On top of that, randomly picking up pulses from the two input binomial RPTs clearly results in the output RPT being binomial too. As noted before, such an RPT has the relative entropy of 1.

This circuit is well known in the literature (see, e.g., Ref. [7]). Because it is theoretically exact and delivers an uncorrelated, maximally random output pulse train, it represents a gold standard for our investigation in this work. For completeness, we note that our previous research referenced in [24] has thoroughly covered other basic arithmetic operations as well: multiplication, division, and subtraction.

For a circuit to be able to perform a certain function, the necessary requirement is that it satisfies the EBC criterion, which for this circuit reads as follows:

(8) $$H\left(\frac{p_{0}+p_{1}}{2}\right)\leq H\left(p_{0}\right)+H\left(p_{1}\right)+H_{int}.$$

This inequality is satisfied for any combination of values of $p_{0}$ and $p_{1}$ because $H_{int}=1$ is a total circuit internal entropy provided by the T random flip-flop (TRFF). Namely, upon each clock (CLK) pulse, the TRFF generates one fresh bit of entropy, thus effectively pushing the whole surface in Figure 4 below or just equal to zero.

In the rest of the paper, given the absence of an exact deterministic circuit for addition, our primary goal is to develop the most precise deterministic circuit through maximizing the entropy utilization of the inputs. The important motivation for this is that, as elaborated in detail in Ref. [15], a maximally random output from an RPC circuit is crucial for its ability to maintain precision in chained or complex calculations. Any autocorrelation of the output RPT will cause errors in subsequent calculations where it is used as an input. As discussed above, any form of autocorrelation in an RPT will manifest itself with the relative entropy being less than 1. Therefore, relative entropy is a good measure of the quality of the output of an RPC circuit.

In the rest of this section, we characterize addition circuits known in the literature and propose new ones.

### 4.1. Analysis of Addition Circuits

#### 4.1.1. Nondeterministic Addition Circuit with MUX

**Figure 6:**
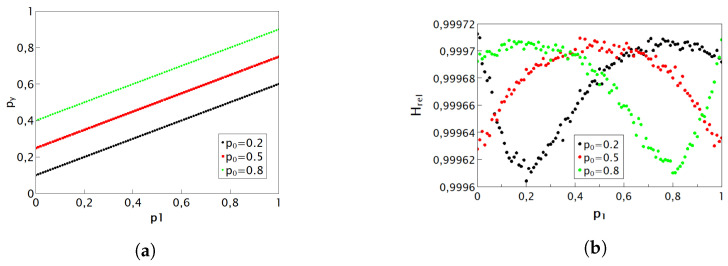
Measurement outcomes of the addition operation using the MUX circuit, considering three fixed values, $p_{0}=0.2,0.5,0.8$, along with varying values of $p_{1}$ (**a**); entropy plot for three preselected values of $p_{0}=0.2,0.5,0.8$, along with different values of $p_{1}$ (**b**). Statistical errors are in the order of $10^{-3}$.

The nondeterministic MUX circuit, shown in Figure 5, is the first known addition circuit that yields theoretically exact results and holds a prominent place in the literature. Consequently, it is valuable to verify its entropy characteristics experimentally. Figure 6a displays measurement results of the addition operation, with data points showcasing a well-expected linear relationship.

A zoom of relative entropy $H_{rel}$ for various combinations of $p_{0}$ and $p_{1}$ is shown in Figure 6b. The statistical error magnitudes mainly reflect the number of output clock periods collected in the experiment. Since this is fixed to $1\times 10^{7}$ throughout all our measurements, the statistical errors are quite small, typically in the order of 0.1%, and negligible with respect to the measured values.

From the results provided, it is evident that a MUX-based circuit indeed delivers accurate addition results. While the relative entropy values all appear to be close to unity, it is crucial to observe that a certain level of dependence on input values seems to persist. Such a dependency should ideally be absent in this circuit, as it is designed for complete randomness and the values should naturally fluctuate in a random manner, deviating more or less from the maximum entropy.

Hence, it is reasonable to assume that this observed dependency remains insignificant within the margins of statistical error. Several factors may contribute to this phenomenon. First, it is possible that the functional dependence arises as a consequence of the numerical methods employed by the programs used to compute relative entropy. Second, there could be variations in the relative entropy of input sequences due to residual autocorrelation within the input signals $p_{0}$ and $p_{1}$. Furthermore, there might be marginal cross-correlations between independent RPTs, such as $p_{0}$ and $p_{0}$, that could be traced back to our imperfect RFFs, although their impact is typically minimal, representing second-order corrections at best. As such, all these assumptions could serve as potential subjects for future research in this field.

#### 4.1.2. Prior Work on Deterministic Addition with MUX

**Figure 7:**
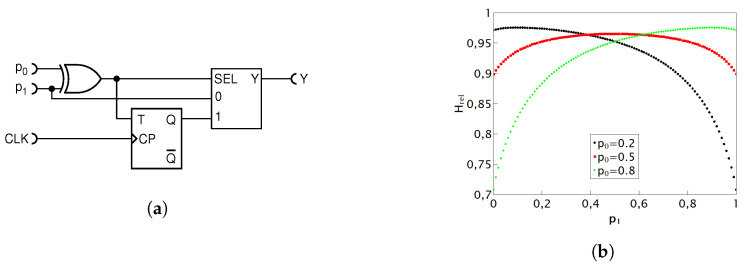
Schematic diagram of the addition circuit introduced in Ref. [19], where $p_{0}$ and $p_{1}$ are the input values and *Y* is the result of adding these input values (**a**); plot of output entropy for the three preselected values of $p_{0}=0.2,0.5,0.8$ as a function of $p_{1}$ (**b**).

Ref. [19] presents a deterministic addition circuit, whose accuracy we will now verify and, for the first time, whose entropy budget we will explore. The circuit is shown in Figure 7 and has been conceived as a deterministic counterpart of the randomized MUX circuit shown in Figure 5 discussed above. This approach is aimed at maximizing the utilization of the existing entropy reservoir while minimizing resource consumption, thereby satisfying the third condition outlined in the introductory section of this chapter for the development of enhanced circuits.

The analysis of the operation of this circuit is not straightforward because the select input (SEL) of MUX is statistically correlated with the values at its inputs. To analyze it, we first note that the pulse probability at the output of the XOR gate equals

(9) $$p_{XOR}=p_{0}+p_{1}-2p_{0}p_{1}$$

by virtue of the laws of probability for independent variables. Next, we note that the input “0” of MUX gets selected when both the inputs $p_{0}$ and $p_{1}$ are equal (i.e., both have a pulse or both do not have a pulse), in which case the state at the input $p_{0}$ is conveyed to the output Y. Thus, the MUX input “0” contributes a pulse only when both inputs are HIGH, which happens with probability $p_{0}p_{1}$. Due to the divide-by-two action of the T flip-flop, the input “1” contributes pulses with probability $p_{XOR}/2$. Thus, the overall probability of pulses at the output Y is given by

(10) $$p_{Y}=p_{0}p_{1}+\left(\frac{p_{0}+p_{1}-2p_{0}p_{1}}{2}\right)=\frac{p_{0}+p_{1}}{2},$$

which is exactly equal to the desired summation function. It is interesting to note that even though this circuit is unsymmetric with respect to its inputs for a deterministic operation, it is symmetric for a stochastic operation. Logically, it looks unsymmetric to its inputs, and the circuit is functionally symmetric. However, if the MUX inputs $p_{0}$ and $p_{1}$ are swapped, the whole addition circuit does not function anymore and outputs gibberish. It turns out that the probability of a pulse at its output Y matches exactly the addition function given in Equation (6).

Unfortunately, the output pulse sequence is heavily self-correlated, and even at its best, the output relative entropy is pretty low, indicating a strong autocorrelation for any combination of input values. If such a circuit is used as an input to other RPC circuits, a great numerical error may occur, as discussed and illustrated in [15].

The measurement results have maintained their precision, and thus, they are not shown. The values of relative entropy, along with their corresponding statistical errors, are presented in Figure 7. As demonstrated in Figure 4, we are aware that an exact deterministic circuit for addition that would produce completely random pulses is entropically not possible. Therefore, the low relative entropy hints at specific correlations within the output sequence. Nevertheless, this circuit remains valuable in pulse computing because it does not rely on an additional entropy source and consistently delivers accurate results.

#### 4.1.3. Equivalent Version of the Deterministic Circuit with MUX

In this section, we present our deterministic modification of the circuit in Figure 7a. It performs the same function, but has a didactic advantage that it can be easily converted into an exact nondeterministic adder, functionally fully equivalent to the one in Figure 5, by simply replacing a deterministic flip-flop with its random counterpart.

**Figure 8:**
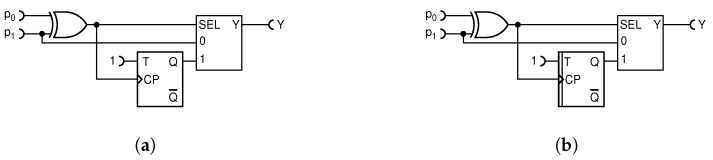
Schematic diagram of the deterministic addition circuit equivalent of the circuit in Figure 7 (**a**); its direct stochastic counterpart (**b**).

Figure 8a illustrates the circuit that is an equivalent version of the previously described addition circuit. It uses exact same logic components, thus having the same hardware cost, while performing the exact same Boolean function. The only distinction lies in how the T flip-flop (TFF) is connected. In this version, a permanent high state is placed at the T input so as to obtain a toggle operation, while the clock input is tied to the output of the XOR gate, thus eliminating the need for the systemic clock (CLK) signal (which in some cases might be a welcome simplification). When the SEL input is LOW, the circuit behaves as the previous one, yielding the pulse probability of $p_{0}p_{1}$. When the SEL input goes HIGH, the output of the TFF will be HIGH at every second time, thus effectively halving the pulse probability at input “1”. Thus, the overall behavior of the circuit is indeed described by Equation (10). As this schematic is equivalent to the first addition circuit, the results are expectedly the same, i.e., precisely identical to the previous circuit. Consequently, they are not reiterated here.

If we now only replace the deterministic TFF with its nondeterministic counterpart T random flip-flop (TRFF), as shown in Figure 8b, the division by two in the second (bracketed) term of Equation becomes nondeterministic. Since the first term $p_{0}p_{1}$ is already nondeterministic, being a product of two nondeterministic processes, the output of the whole circuit is also nondeterministic. This circuit is an equivalent of the gold standard circuit of Figure 5, but using a bit more of hardware, namely, one extra XOR gate.

This display of the two equivalent versions of the same deterministic circuit, as well as of the two equivalent nondeterministic adders, serves as a noteworthy illustration of the versatility within RPC and the potential it unlocks by carefully combining logic gates to obtain the desired stochastic function.

#### 4.1.4. Simplest Deterministic Addition Circuit with MUX

We start the process of conceiving our deterministic adder by noting that in the gold standard nondeterministic adder circuit, shown in Figure 5, the critical requirement is that the select input (SEL) of the MUX input is supplied by a stream of random bits with an equal probability of ones and zeros = at each CLK period. In fact, this is the only relevant requirement for this adder to work flawlessly. As noted before, such a stream of bits features the maximum entropy of 1 and is just enough to balance the entropy budget for any pair of input values.

**Figure 9:**
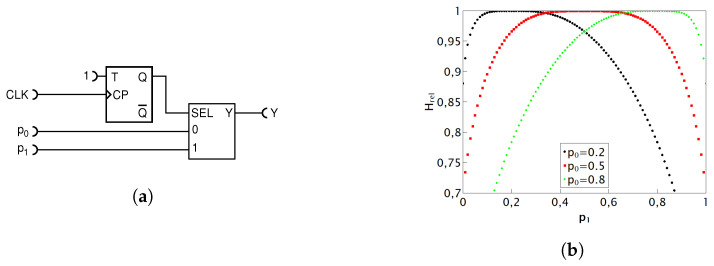
The simplest deterministic MUX-based addition circuit (**a**); its entropy plot for the three preselected values of $p_{0}=0.2,0.5,0.8$ as a function of $p_{1}$ (**b**).

Arguably the simplest deterministic counterpart of the nondeterministic MUX adder circuit is obtained by replacing the random T flip-flop with an ordinary deterministic T flip-flop, as shown in Figure 9a.

The T flip-flop samples each input RPT with the probability of exactly one-half, sending pulses appearing on odd time intervals from the first input and pulses appearing on even time intervals from the second input, to the output Y. Therefore, the pulse probability at the output of the circuit is exactly equal to the desired function in Equation (6). Furthermore, since input RTPs are statistically independent and random by definition, one is tempted to conclude that the output RPT, so composed, would be perfectly random. However, as noted in our previous work [23], the waiting time distribution of pulses, when exactly every second is omitted, shifts from the desired geometric distribution (characteristic of the binomial random process) to the Erlang distribution and causes strong autocorrelation. Our experiment reveals that the relative entropy at the output of this circuit varies a lot. It has regions of very high, near-unity relative entropy, but it also has regions where it fails miserably. Failure is especially pronounced when one input probability is low and the other high, because then the deterministic toggling of the T flip-flop creates an almost alternate pattern of zeros and ones that has a strong (negative) autocorrelation and, as such, is unusable for further processing by RPC circuits. When compared with the performance of the previous circuit shown in Figure 7, one could say that it is inferior for general use, but also that it performs better for a certain range of input probabilities. Notably, as can be seen from Figure 9b, whenever the two input probabilities are close to each other, i.e., do not differ by about more than 0.1, this circuit outputs the relative entropy very close to unity and, in that case, significantly outperforms the previous circuit. Thus, depending on the application, one or the other circuit might be preferred.

#### 4.1.5. First Improved Deterministic Adder

Given that a deterministic circuit for addition can provide accurate results for the addition operation while relative entropy tends to be low, indicating specific correlations in the output signal, we endeavor to design a circuit that maintains the computational precision while generating a more random output sequence to bring the values of relative entropy closer to unity.

After deciding that the input RPTs will be symmetrically connected to the inputs “0” and “1” of a MUX in order to achieve a symmetry between them, we start the process of conceiving our deterministic adder by realizing that a stream of random bits with equal probability of ones and zeros should supply the SEL input of the MUX. A sought-for deterministic adder would perform as good as one could mimic such a stream within it. By definition, in a deterministic adder, the only available entropy is that of the two input RPTs. It is well known in the art that XOR-ing two weakly random streams of bits produces a third stream that is more random than any of the two (or equal to the one that is more random). By XOR-ing the two input RPTs, one obtains a new stream with the pulse probability $p_{0}+p_{1}-2p_{0}p_{1}$, as already explained in Equation (9). This is generally not equal to 1/2, except for a few special choices of the input probabilities $p_{0}$ and $p_{1}$, and therefore is not suitable for a direct drive of the SEL input of the MUX.

**Figure 10:**
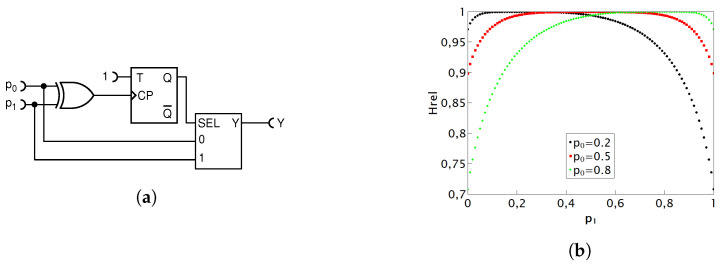
The improved deterministic MUX-based addition circuit (**a**); the entropy plot for the three preselected values of $p_{0}=0.2,0.5,0.8$ as a function of $p_{1}$ (**b**).

To remedy that, we use the simplest possible solution and divide the stream by two using a toggle T flip-flop, as shown in Figure 10a. This circuit resembles the simplest nondeterministic circuit in Figure 9, except that the toggle flip-flop is not driven by the periodic clock CLK, but by a partially random stream of pulses. It clearly maintains the exact function of Equation (6), because the probability of the T flip-flop being in a HIGH state is equal to it being in a LOW state, but how well does it perform with respect to the relative entropy of the output?

The relative entropy values, shown in Figure 10b, reach quite close to unity in approximately 50% of the input value combinations $p_{0}$ and $p_{1}$, whereas the previous art in Figure 7 fails to reach the value of one in any scenario. Consequently, this deterministic circuit is a more preferable choice for combining various operations to tackle a more intricate computation.

#### 4.1.6. Second Improved Deterministic Adder

**Figure 11:**
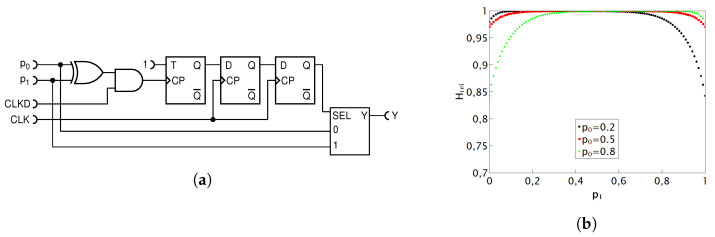
The second improved deterministic addition circuit (**a**); its entropy plot for three preselected values of $p_{0}$ as a function of $p_{1}$ (**b**).

While the previously described circuit provides precise results and high values of relative entropy, we now contemplate whether it is possible to further approach unity for the relative entropy of the output, especially in the parameter space where it was not achieved by previous circuits. We note that the random drive to the T flip-flop in the previous circuit in Figure 10a, and consequently its output state, is correlated with the input pulses because the same pulses affect the SEL input simultaneously with their arrival at MUX inputs. To address this, an enhancement was introduced to increase the randomness in selecting output pulses within the MUX. This was achieved by introducing a delay of two clock periods to the output from the XOR gate. The circuit is depicted in Figure 11a.

The D flip-flop is usually used for signal delays and functions optimally when the D input is not transitioning between states to prevent unstable readings. To ensure this, we introduced an immediate delay of 1/4 CLK period to the signal from the XOR gate. This ensured that state transitions occurred within the pulse’s stability period at the D input. After the first D flip-flop, the signal from the T flip-flop is delayed by a total of one full period. Subsequently, the second D flip-flop adds an extra full period of delay, resulting in a total delay of two full CLK periods at the output of the second D flip-flip compared with the output from the XOR gate. This means that the results of XOR-ing are now two periods behind the input RPTs and are thus well statistically isolated. This technique of statistical isolation is well known in the art and is explained in Ref. [15]. Experimentally, we noted that adding more delay does not have influence (or has a very minimal one) on the further improvement of relative entropy, but it obviously does cost additional resources. Therefore, we conclude that this circuit is optimal regarding the improvement strategy being used.

In this circuit, the precision of the addition operation results has been maintained, but the relative entropy of the output RPT has dramatically improved. From Figure 11b, we can observe that the values of relative entropy have further approached unity with minimal additional resource consumption, while still upholding the computational accuracy of the addition operation. This circuit represents the best deterministic addition circuit known thus far. That being said, we leave for further study the question of how far a deterministic adder circuit can be improved and at what hardware and energy cost.

Before ending this presentation, we wish to present one last result in order to point out that not only deterministic but also perfect nondeterministic adders may be improved based on the notion of entropy.

#### 4.1.7. A Nondeterministic Adder with Minimal Internal Entropy Usage

**Figure 12:**
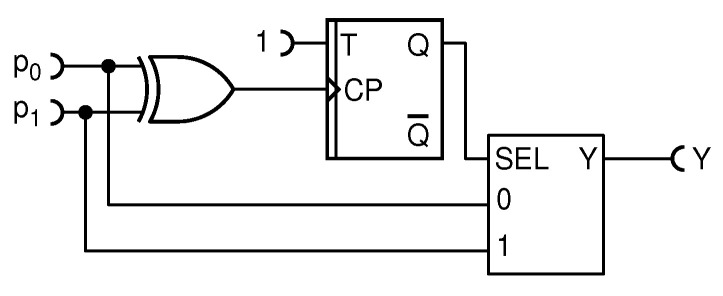
Schematic diagram of the resource-improved addition circuit.

Observing the gold standard nondeterministic adder in Figure 5, one can note that it uses a lot of internal entropy: one random bit per CLK pulse. However, is all that entropy necessary? When both the inputs $p_{0}$ and $p_{1}$ are of same state (HIGH or LOW), then the state of the SEL input of the MUX is irrelevant because whichever is the state of the input(s), it will be conveyed to the output Y. Therefore, no additional internal entropy is needed. On the contrary, when states are not the same, then SEL must randomly select which input to convey to the output Y. In such a case, a fresh, new bit of entropy is mandatory. The addition circuit that requests a random bit from the internal entropy source (TRFF) only when needed is shown in Figure 12.

This circuit is functionally equivalent to the gold standard circuit shown in Figure 5. The achieved entropy savings depends on the input probabilities. For example, if at least one input RPT has pulse probability close to 1/2, then it will randomly coincide with the other with a probability of 1/2, thus reducing the required entropy by a factor of 2 with respect to the gold standard circuit. If both input probabilities are LOW (or HIGH), the input RPTs will coincide most of the time and a very little internal entropy will be needed. Finally, in the area of red tongues shown in Figure 4, where one input pulse probability is low and the other is high, output randomness comes almost solely from the internal TRFF and the required rate of entropy is high, but lower than in the original circuit. Thus, the entropy savings is anywhere between moderate and dramatic. This could be of interest in entropy-starving situations, such as when entropy is served from a reservoir of limited capacity or for energy or hardware reduction. It is interesting to note that this circuit may be derived from our first improved deterministic circuit, shown in Figure 10a, by simply replacing the TFF with a TRFF. This effectively converts the worst deterministic adder considered here into the theoretically perfect adder with the lowest entropy consumption known so far. This is yet another example of how a small, but educated, modification of an RPC circuit can yield an improvement or an optimization.

## 5. Discussion and Conclusions

Research in the field of electronic circuits for mathematical operations in random pulse computing is driven by the need to build a universal programmable random pulse computer (RPC). Such a computer would operate on principles reminiscent of the neural systems found in living organisms, where electrical impulses in the brain correspond to logical pulses. In this study, using the information-theoretic tools, we delved into the examination of the inner workings, different optimizations, and overall performance of several deterministic and nondeterministic electronic addition circuits.

In the art of RPC, both deterministic and nondeterministic computation circuits are used. The entropy budget criterion (EBC) represents the minimum requirement for a given circuit to be able to perform a given mathematical function. In practice, it means that the circuit functions imperfectly in some way: it may be imprecise in calculating the target function, or it may produce a low relative entropy at its output or both. For example, for the deterministic adder of Ref. [19], shown in Figure 7, we are able to immediately predict that it cannot be exact because, by virtue of the EBC, the required mathematical operation necessitates an internal entropy source that is not present in the mentioned circuit; therefore, it does not satisfy the EBC. By the strict probability theory analysis, we have shown that its output probability in fact does exactly match the required mathematical operation (addition). However, we have also found, by experiment, that its output relative entropy is quite low, which means that if used as an input to the other RPC circuits, it will cause further entropy degradation and computational inaccuracy, an effect well recognized in the field, as discussed in the Introduction. Namely, any RPC circuit works under the assumption of perfectly random input RPTs. Furthermore, upon closer inspection, we noted that less entropy is used in this circuit than is available from inputs, which gave us a clue on how to improve on it.

That led us to propose three improved deterministic addition circuits, presented here for the first time. They all compute exactly but offer different levels of hardware–entropy trade-offs. The simplest one, shown in Figure 9, requires the minimum count of logic gates and offers a near-maximum output relative randomness when the two input numbers (probabilities) are within ±0.1 from each other. The first improved deterministic circuit, shown in Figure 10, at a cost of one additional XOR gate, significantly widens the input range within which its output entropy is near maximum, and it does not require the CLK signal, thus putting fewer requirements on signal routing in an RPC processor. The second improved deterministic circuit, shown in Figure 11, has a much larger range of input values for which it outputs very high relative entropy, but at a certain cost in hardware and more complicated clocking.

The rationale behind the search for a deterministic adder is that it does not require a potentially hardware-expensive source of entropy, such as a TRFF. On the other hand, the drawback of a deterministic adder is its nonperfectly random output, which can cause computational errors in the downstream RPC circuits. Nevertheless, in some applications, deterministic adders may offer an acceptable trade-off between the precision and hardware cost.

Finally, we proposed an improved nondeterministic, perfect adder, shown in Figure 12, which uses less entropy from the internal entropy source than the circuit known so far—indeed, it uses only as much entropy as theoretically required. Furthermore, unlike the previous art, it does not need the CLK signal. Such a circuit would be valuable in an environment where the generation of entropy is resource expensive (e.g., in terms of hardware, energy, etc.), and therefore, entropy may not be wasted.

In conclusion, we believe that advancements in RPC circuits, enabled by the tools exercised here, namely, the EBC and relative entropy, have the potential to enable the seamless integration of various mathematical operations, leading to more precise solutions for a wide range of problems, including numerical computing, image processing, and neural network techniques. This competitive advantage could position the RPC paradigm ahead of other computing approaches, with possible applications spanning from artificial intelligence to a deeper understanding of neurons and the workings of the human brain.

## Data Availability

The data that support the findings of this study are available from the corresponding author, MS, upon reasonable request.

## References

[1] (2021). Croatian Encyclopedia, Online Edition.

[2] Von Neumann J. (1956). Probabilistic logics and synthesis of reliable organisms from unreliable components. Autom. Stud..

[3] Lawlor R.C. (1971). Computer Utilizing Random Pulse Trains. U.S. Patent.

[4] Ribeiro S.T. (1967). Random-Pulse Machines. IEEE Trans. Electron. Comput..

[5] Gaines B.R. (1969). Stochastic Computing Systems. Adv. Inf. Syst. Sci..

[6] Alaghi A., Hayes J.P. (2013). Survey of stochastic computing. ACM Trans. Embed. Comput. Syst. (TECS).

[7] Alaghi A., Qian W., Hayes J.P. (2018). The promise and challenge of stochastic computing. IEEE Trans. Comput.-Aided Des. Integr. Circuits Syst..

[8] Fick D., Kim G., Wang A., Blaauw D., Sylvester D. Mixed-Signal Stochastic Computation Demonstrated in an Image Sensor with Integrated 2D Edge Detection and Noise Filtering. Proceedings of the IEEE 2014 Custom Integrated Circuits Conference.

[9] Qian W., Li X., Riedel M.D., Bazargan K., Lilja D.J. (2011). An architecture for fault-tolerant computation with stochastic logic. IEEE Trans. Comput..

[10] Alaghi A., Hayes J.P. A spectral transform approach to stochastic circuits. Proceedings of the IEEE 30th International Conference on Computer Design.

[11] Luong T.-K., Nguyen V.-T., Nguyen A.-T., Popovici E. Efficient architectures and implementation of arithmetic functions approximation based stochastic computing. Proceedings of the 2019 IEEE 30th International Conference on Application-specific Systems, Architectures and Processors (ASAP).

[12] Qin Z., Qiu Y., Zheng M., Dong H., Lu Z., Wang Z., Pan H. (2020). A Universal Approximation Method and Optimized Hardware Architectures for Arithmetic Functions Based on Stochastic Computing. IEEE Access.

[13] Ting P.-S., Hayes J.P. Isolation-based decorrelation of stochastic circuits. Proceedings of the 2016 IEEE 34th International Conference on Computer Design (ICCD).

[14] Qian W., Backes J., Riedel M. (2009). The Synthesis of Stochastic Circuits for Nanoscale Computation. Int. J. Nanotechnol. Mol. Comput..

[15] Liu Y., Parhi K.K. (2017). Computing Polynomials Using Unipolar Stochastic Logic. J. Emerg. Technol. Comput. Syst..

[16] Lee V.T., Alaghi A., Ceze L. Correlation manipulating circuits for stochastic computing. Proceedings of the Design, Automation and Test in Europe Conference and Exhibition.

[17] Parhi M., Riedel M.D., Parhi K.K. Effect of bit-level correlation in stochastic computing. Proceedings of the 2015 IEEE International Conference on Digital Signal Processing (DSP).

[18] Liu S., Han J. Energy efficient stochastic computing with sobol sequences. Proceedings of the Design, Automation Test in Europe Conference Exhibition.

[19] Lee V.T., Alaghi A., Hayes J.P., Sathe V., Ceze L. Energy-efficient hybrid stochastic-binary neural networks for near-sensor computing. Proceedings of the Design, Automation and Test in Europe Conference and Exhibition.

[20] Joe H., Kim Y. (2019). Novel Stochastic Computing for Energy-Efficient Image Processors. Electronics.

[21] Schober P., Estiri S.N., Aygun S., Jalilvand A.H., Najafi M.H., TaheriNejad N. (2023). Stochastic Computing Design and Implementation of a Sound Source Localization System. IEEE J. Emerg. Sel. Top. Circuits Syst..

[22] Liu Y., Liu S., Wang Y., Lombardi F., Han J. (2020). A Survey of Stochastic Computing Neural Networks for Machine Learning Applications. IEEE Trans. Neural Netw. Learn. Syst..

[23] Stipčević M. (2016). Quantum random filp-flop and its applications in random frequency synthesis and true random number generator. Rev. Sci. Instrum..

[24] Stipčević M., Batelić M. (2022). Entropy considerations in improved circuits for a biologically-inspired random pulse computer. Sci. Rep..

[25] Keshavarzian P. (2023). A 3.3-Gb/s SPAD-based quantum random number generator. IEEE J. Solid-State Circuits.

[26] Stipčević M. (2009). Active quenching circuit for single-photon detection with Geiger mode avalanche photodiodes. Appl. Opt..

[27] Terrasic DE0-Nano Datasheet. https://www.terasic.com.tw/cgi-bin/page/archive.pl?Language=English&CategoryNo=139&No=593&PartNo=4#contents.

[28] Koch C., Bernander O., Douglas R.J. (1995). Do neurons have a voltage or a current threshold for action potential initiation?. J. Comput. Neurosci..

[29] Ulku A.C., Bruschini C., Antolovic I.M., Charbon E., Kuo Y., Ankri R., Weiss S., Michalet X. (2019). A 512 × 512 SPAD Image Sensor with Integrated Gating for Widefield FLIM. IEEE J. Sel. Top. Quantum Electron..

